# Herbal Medicines for Post-Acute Sequelae (Fatigue or Cognitive Dysfunction) of SARS-CoV-2 Infection: A Phase 2 Pilot Clinical Study Protocol

**DOI:** 10.3390/healthcare10101839

**Published:** 2022-09-22

**Authors:** Kyung Hwan Jegal, Jiwon Yoon, Sanghyun Kim, Soobin Jang, Young-Hee Jin, Jun-Hwan Lee, Sun-Mi Choi, Tae Hun Kim, Sunoh Kwon

**Affiliations:** 1 Digital Health Research Division, Korea Institute of Oriental Medicine, Daejeon 34054, Korea; 2 College of Korean Medicine, Daegu Haany University, Gyeongsan 38610, Korea; 3 Korean Medicine Data Division, Korea Institute of Oriental Medicine, Daejeon 34054, Korea; 4 Korean Medicine Application Center, Korea Institute of Oriental Medicine, Daegu 41062, Korea; 5 Korean Medicine Science Research Division, Korea Institute of Oriental Medicine, Daejeon 34054, Korea; 6 UST KIOM School, University of Science and Technology (UST), Daejeon 34113, Korea; 7 Korean Medicine Clinical Trial Center, Korean Medicine Hospital, Kyung Hee University, Seoul 02447, Korea; 8 Korean Medicine Convergence Research Division, Korea Institute of Oriental Medicine, Daejeon 34054, Korea

**Keywords:** COVID-19, SARS-CoV-2, long COVID, sequelae, fatigue, cognitive dysfunction, herbal medicine, Korean Medicine, Bojungikgi-tang, Kyungok-go, Cheonwangbosim-dan

## Abstract

Long-term sequelae refer to persistent symptoms or signs for >6 months after SARS-CoV-2 infection. The most common symptoms of sequelae are fatigue and neuropsychiatric symptoms (concentration difficulty, amnesia, cognitive dysfunction, anxiety, and depression). However, approved treatments have not been fully established. Herbal medicines are administered for 12 weeks to patients who continuously complain of fatigue or cognitive dysfunction for >4 weeks that only occurred after COVID-19 diagnoses. Based on the Korean Medicine syndrome differentiation diagnosis, patients with fatigue will be administered Bojungikgi-tang or Kyungok-go, whereas those with cognitive dysfunction will be administered Cheonwangbosim-dan. Results could support evidence that herbal medicines may mitigate fatigue and cognitive dysfunction caused by COVID-19. Furthermore, by investigating the effects of herbal medicines on changes in metabolite and immune response due to COVID-19, which may be responsible for sequelae, the potential of herbal medicines as one of the therapeutic interventions for post-acute sequelae of SARS-CoV-2 infection can be evaluated. Therefore, the effects of herbal medicine on fatigue and cognitive dysfunction sequelae due to COVID-19 will be elucidated in this study to provide an insight into the preparation of medical management for the post-acute sequelae of SARS-CoV-2 infection.

## 1. Introduction

Due to the global Coronavirus Disease 2019 (COVID-19) pandemic, medical and research capabilities have concentrated on the development of vaccines for the prevention of infectious diseases, blocking the spread of infection, and treatment of acute infectious conditions. However, life-threatening acute infections, including post-acute sequelae of SARS-CoV-2 infection, have been widely reported. A recent study on the follow-up observation of 1250 patients with severe COVID-19 who were hospitalized in 38 hospitals in Michigan, USA, reported that many COVID-19 survivors were unable to perform their normal activities due to various physical and mental sequelae after discharge. They reported difficulties in performing activities of daily life that lead to reduced working hours or complete unemployment [1]. Symptom management after a complete recovery from acute SARS-CoV-2 infection currently requires attention.

Fatigue is the most frequently reported symptom among the COVID-19 sequelae. More than half of survivors had fatigue for at least 6 weeks; however, the relationship between severity of acute COVID-19 and fatigue has not been confirmed, which might suggest that even survivors of mild COVID-19 can experience fatigue that lasts for a considerable time. Notably, more than half of those who have recovered from COVID-19 complain of fatigue regardless of symptom severity [2]. Further, cognitive dysfunction, also known as “brain fog”, is reported in a large number of patients who have recovered from COVID-19. Researchers at Imperial College London reported in an intelligence test they conducted that 84,285 people who had recovered from COVID-19 showed lower cognitive abilities than those who were not infected. In particular, it was reported that the more severe the COVID-19 symptoms, the greater the sequelae, and brain clouding also occurred in participants who had recovered for several months. Those who had recovered from COVID-19 scored lower verbal ability, logical ability, spatial perception ability, concentration, and emotional regulation ability than non-infected people, and the brain age of patients who were treated in an intensive care unit or used a ventilator, was at the maximum. Such forgetfulness (decrease in cognitive ability) decreased daily life and work ability, and was commonly experienced by patients with severe symptoms in the acute stage as well as recovered patients who had experienced milder symptoms and who had not received inpatient treatment [3].

As for these sequelae of COVID-19, accurate identification of pathological mechanisms of each symptom is an urgent medical problem; nonetheless, an appropriate treatment strategy is not yet established [4]. In this context, we will try to access the potential effects of widely used herbal medications for patients with fatigue and cognitive dysfunction after recovering from COVID-19, and to test the feasibility of this clinical study design for future clinical trials with sufficient sample size on these symptoms in the future.

## 2. Methods

### 2.1. Objectives

This study is a phase 2 preliminary clinical trial to evaluate the safety and effectiveness of commercially available herbal medicines for fatigue or cognitive dysfunction after recovering from COVID-19. It also aims to provide basic evidence for future large-scale clinical trials by evaluating the feasibility of the study design and interventions. The primary objective is to investigate the effects of 12-week herbal medication on the treatment success rate in patients with fatigue or cognitive dysfunction after recovering from acute COVID-19 infection. The secondary objective is to evaluate the feasibility of the study design, such as patient recruitment rate and dropout rate during the trial. Moreover, the effects of herbal medicines on immune responses related to COVID-19 and on metabolites related to fatigue and cognitive dysfunction will be assessed.

### 2.2. Study Design

**Figure 1:**
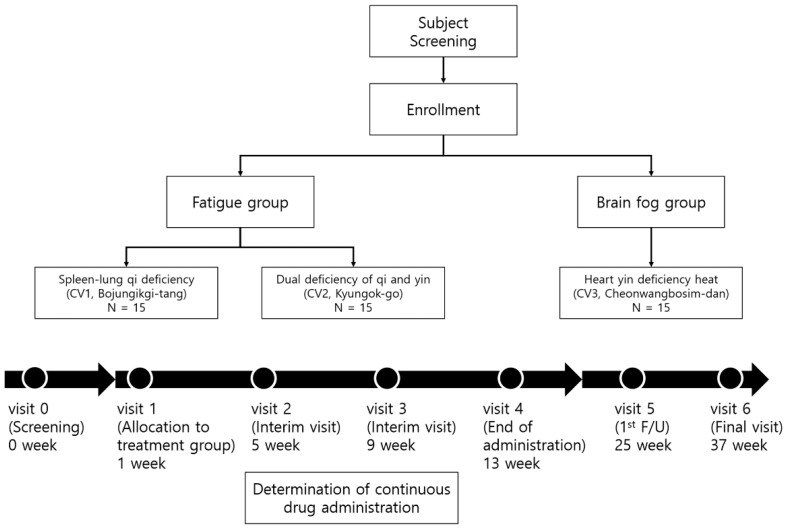
Flow diagram and schedule.

Written informed consent will be obtained from all patients before participating in this study. We will recruit patients with fatigue or cognitive symptoms and three Korean Medicine (KM) herbal medications will be prescribed to each patient based on the syndrome differentiation based on the symptom type. Patients will visit once per month during a 12-week medication period and then will visit for the examination of symptoms, a blood chemistry check, and immunological tests every 12 weeks over 24 weeks. The flow diagram and study schedule are shown in Figure 1 and Table 1.

### 2.3. Sample Size

The current study is a phase 2 preliminary clinical study, thus we will recruit 45 patients without sample size calculations. Fifteen patients will be assigned to each of the three intervention groups. The results from this study will be used to calculate the sample size for further confirmatory clinical trials.

### 2.4. Participants

#### 2.4.1. Inclusion Criteria

The inclusion criteria are as follows:

- Those aged >19 years and have been diagnosed with COVID-19 and recovered. To confirm the COVID-19 diagnosis and recovery, medical history will be examined by reviewing the submitted medical records about the diagnosis, hospitalization, and certificate of SARS-CoV-2 negative or conduct COVID-19 antibody test using the COVID-19 IgM/IgG Plus Test kit (Sugentech, Inc., Daejeon, Korea) approved by the Korean Ministry of Food and Drug Safety. If submission of medical records is possible, the antibody test can be skipped.
- Those who are continuously complaining of fatigue or cognitive dysfunction for >4 weeks that they did not experience before their COVID-19 diagnosis.
- Those who scored a total of >76 points on the Checklist Individual Strength (CIS).
- Participants do not have any problems with overall cognitive function and must be capable of providing written informed consent to participate in the study.

#### 2.4.2. Exclusion Criteria

The following exclusion criteria are considered:

- Those who have been diagnosed with diseases that can cause fatigue (cancer, sleep disturbance, chronic hepatitis, liver cirrhosis, chronic renal failure, tuberculosis, asthma, multiple sclerosis).
- Those who have been diagnosed with diseases that can cause brain fog (cerebral hemorrhage, cerebral infarction, brain tumor, Parkinson’s disease, epilepsy, major depressive disorder, bipolar affective disorder, schizophrenia, delusional disorder, or dementia).
- Those who have medical problems that may affect the intake or absorption of drugs (dysphagia, clinically serious digestive disorders, galactose intolerance, Lapp lactase deficiency, or glucose-galactose malabsorption).
- Those currently with or have a medical history of allergy to clinical trial drugs (herbal medicines).
- Those who have been diagnosed with liver or kidney disease, or have abnormal levels in blood tests (exceed 3 times the upper limit of normal aspartate aminotransferase (AST), alanine aminotransferase (ALT), blood urine nitrogen (BUN), or creatinine level).
- Pregnant, lactating, and fertile women who have a pregnancy plan.
- Those who have participated in other clinical trials within 30 days of participating in this study.
- Those who are judged to be inappropriate for the clinical trial by the researchers due to clinically significant psychiatric symptoms, physical conditions, laboratory findings or other medical states.

#### 2.4.3. Withdrawal and Discontinuation Criteria

The reasons for withdrawal and discontinuing herbal medication or observation shall be documented. The patients’ withdrawal criteria are as follows:

- Acute reaction (allergy, hypersensitivity, and others) to herbal medicine.
- A violation of the inclusion/exclusion criteria is confirmed during the trial.
- No longer possible to administer or make observations due to unexpected diseases or accidents.
- No longer possible to administer or make observations due to severe adverse events or adverse drug reaction.
- The patient is pregnant.
- Withdrawal of consent by patients or their agents.
- It is judged by researchers that the trial is difficult to continue.

### 2.5. Recruitment

The present study aims to recruit 45 participants from Kyung Hee University Korean Medicine Hospital through advertisements on public transport or promotions in COVID-19 patient treatment facilities (e.g., National Medical Center) and related disease cohorts.

### 2.6. Blinding and Treatment Allocation

Due to the design characteristics of this clinical study without a control group, randomization, allocation concealment, and blinding will not be implemented. The investigator shall assign each patient a unique patient number at visit 0 (V. 0). To allocate patients to each herbal medicine group, the KM syndrome differentiation will be conducted by an internal medicine specialty Korean Medicine doctor (KMD) with a clinical experience of >10 years. According to the main sequelae symptoms after COVID-19, patients will be allocated to the fatigue or cognitive dysfunction group and then assigned to the appropriate herbal medicine intervention group based on each sub-syndrome differentiation pattern. The considerations for syndrome differentiation are shown in Table 2.

### 2.7. Intervention

Based on the syndrome differentiation diagnosis, three different herbal medications will be prescribed to each patient for up to 12 weeks. Among the patients with fatigue, the pattern/syndrome of lung-spleen qi deficiency group will be administered Bojungikgi-tang (CV1, Kracie Bojungikgitang Extract Fine Granule, Kolmar Pharma, Korea), and the syndrome of dual deficiency of qi and yin group will be administered Kyungok-go (CV2, Kyungbang Kyungokgo, KBPharm, Korea). The cognitive dysfunction group will be administered Cheonwangbosim-dan (CV3, Soonsimhwan, Hanpoong Pharm, Korea). Bojungikgi-tang (CV1) will be administered one sachet (3.75 g) twice a day before or in between meals. Kyungok-go (CV2) will be administered one sachet (20 g) twice a day in the morning and evening before or in between meals. Cheonwangbosim-dan (CV3) will be administered one sachet (one dose) once a day in between meals. The evaluation for the continuation of herbal medicine administration will be conducted at visits 2 and 3. If visual analog scale (VAS, 0–100) for fatigue or cognitive dysfunction at the current visit is improved by <50% of VAS at the previous visit, and the patients want to take herbal medicine, the same herbal medicine as previously prescribed will be re-administered for 4 weeks. For this evaluation, the symptom improvement rate at the current visit (VAS score of the current visit/VAS score of the previous visit (%)) and whether the patients want to continue the administration of herbal medicines will be investigated.

### 2.8. Outcomes

#### 2.8.1. Primary Outcome

As the primary outcome, the treatment success rate after herbal medications for up to 12 weeks for fatigue and cognitive dysfunction in patients who have recovered from COVID-19 will be evaluated. VAS (0–100) for fatigue or cognitive dysfunction before (visit 1) and after administration of herbal medicines (visit 4) will be assessed. If VAS is >15 points, it is defined as treatment success. The frequency or ratio of treatment success patients will be presented, based on the fatigue and cognitive dysfunction groups, respectively.

#### 2.8.2. Secondary Outcome

##### Medication Adherence

Medication adherence will be monitored by counting the number of returned and taken medications at each visit. In the case of the number of returned drugs and number of taken drugs being unmatched with the number of prescribed drugs, the reason for this shall be recorded in the case report form. Medication adherence is calculated as follows:

$$Medication\;adherence\;\left(\%\right)=\frac{The\;number\;of\;taken\;drugs}{The\;number\;of\;prescribed\;drugs\;in\;the\;duration\;*}\times 100$$

* The number of prescribed drugs in the duration means the number of drugs should be taken from the time of initial administration to before the time of returning the untaken drugs.

The final medication adherence will be evaluated at 12 weeks after the initial administration (visit 4 or end point of medication), and the sum of the number of taken herbal medicines divided by the sum of the number of prescribed herbal medicines in the duration will be calculated and collected at visits 2, 3, and 4. Mean, standard deviation, median, minimum, and maximum value of the final medication adherences (%) for all study patients will be represented for each herbal medicine group, and multiple comparison analyses will be conducted among groups.

##### Checklist Individual Strength (CIS)

CIS is an indicator for evaluating fatigue in the last 2 weeks, and will be accessed by patients themselves using a questionnaire as instructed by the principal investigator or other researchers at visits 2, 3, 4, 5, and 6. Each patient will answer the CIS questionnaire after receiving sufficient explanation about its purpose and method from the clinical investigator [5]. The total CIS score will be compared among the intervention groups for each visit.

##### Chalder Fatigue Scale (ChFS) and Subscale

The ChFS will be evaluated by using the numeric rating scale 100 (range 0–100) mm following the Korean version of ChFS. The total score and subscale for physical- (No. 1–7) and mental health (No. 8–11) will be recorded at visits 2, 3, 4, 5, and 6 [6]. The total score and subscale scores will be calculated and compared among groups for each visit.

##### EQ-5D-5L

EQ-5D is a standardized measure of health-related quality of life. EQ-5D classifies the health state profile as five levels in five dimensions. The five dimensions are mobility, self-care, usual activity, pain/discomfort, and anxiety/depression. The five levels in each dimension are classified as: (1) no problems, (2) slight problems, (3) moderate problems, (4) severe problems, and (5) unable to (mobility, self-care, usual activities), extreme (pain/depression) or extremely (anxious/depressed). EQ-5D scores will be calculated at visits 2, 3,4, 5 and 6, and compared among groups for each visit.

##### Pittsburgh Sleep Quality Index (PSQI)-K

The PSQI-K is a self-reported questionnaire for evaluating sleep quality that consists of questionnaires assessing seven components about sleep quality including subjective sleep quality, sleep latency, sleep duration, habitual sleep efficiency, sleep disturbances, use of sleeping medication, and daytime dysfunction. The global score is calculated by the sum of the score ranging from 0 to 21. Global scores will be assessed at visits 2, 3, 4, 5, and 6 and compared among groups for each visit.

##### Korean-Montreal Cognitive Assessment (K-MoCA)

The K-MoCA is a simple cognitive function assessment tool and is more sensitive than a mini-mental state exam (MMSE) in assessing cognitive decline. K-MoCA consists of visuospatial/executive (5 points), naming (3 points), attention (6 points), language (3 points), abstraction (2 points), orientation (6 points) and delayed recall (5 points). The maximum score is 30 points, and the normal range is >23 points [7]. Total scores will be calculated at visits 2, 3,4, 5, and 6 and compared among groups for each visit.

##### Cognitive Failure Questionnaire (CFQ)

The CFQ is an evaluation tool for cognitive function by measuring slip and error of perception, memory, and motor functioning in daily life, consisting of 25 individual items, and each item is evaluated by frequency of mistakes, rating from 0 (never) to 4 (very often). The total score is calculated by the sum of the rating of 25 items, yielding a score from 0–100. Forgetfulness, distractibility, and false triggering can be assessed through CFQ. CFQ will be assessed at visit 6, and compared among multiple groups [8].

##### Beck’s Depression Inventory (BDI)

BDI will be assessed to evaluate depression and its severity after COVID-19 or recovery. BDI consists of 21 items in four domains of cognitive, emotional, motivational, and physical symptoms of depression. Each item is rated on a scale of 0 to 3, and the total score of the items is ranged 0–63 points, with higher scores indicating more severe symptoms of depression. BDI will be assessed at visits 2, 3, 4, 5, and 6, and compared among multiple groups [9].

##### Digit Span Test in Korean-Wechsler Adult Intelligence Scale (WAIS)

The WAIS, the most widely used intelligence test tool in clinical practice, is used to measure cognitive impairment changes in cognitive dysfunction. The digit span test, a subset of WAIS, is a well-validated and convenient tool to measure verbal short-term memory and working memory capacity. It consists of digits forward (DF) and digits backward (DB) components, which require patients to repeat a series of spoken digits in either forward (usually 3–9 digits) or reverse order (2–8 digits), respectively. In case of false response in a row, the test is discontinued. The DF score is the number of digits of the largest number correctly spoken by the patient, and the DB score is the number of digits of the largest number correctly repeated by the patient. A score of DF, DB, and DF minus DB is calculated. Higher DF and DB scores indicate better short-term memory. In cases of DF minus DB scores of >5 points, the working memory is determined as abnormal. The digit span test will be assessed at visits 4, 5, and 6, and compared among multiple groups [10].

##### Korean-Boston Naming Test-15 (K-BNT-15)

The BNT is widely used to determine changes in language proficiency based on cognitive impairments, a validated test for visual confrontation naming abilities. The 60-item version of BNT contains a 60-line drawing of objects to naming response, and requires 15–30 min to test. The BNT-15 is the shortened version of BNT and simplified to test the administration and scoring process in a short time [11]. In the current study, the Korean version of BNT-15 will be used to examine language function changes based on cognitive dysfunction [12]. For this test, “Dragon”, “Tadpole”, “Cobweb”, “Snowman”, “Boots”, “Turtle Ship”, “Icicle”, “Camel”, “Thimble”, “Extinguisher”, “Mermaid”, “Dinosaur”, “Stroller”, “Toshi (armband)”, and “Starfish” are shown in sequence and, if it is the correct answer, rates 1 point; otherwise, it rates 0 points. Total score is added up to calculate the K-BNT-15 score. The K-BNT-15 test will be assessed at visits 4, 5, and 6, and compared among multiple groups.

##### Analysis of Patient Recruitment Rate, Enrolment Rate, Dropout Rate, and Reasons for Dropout for Feasibility Evaluation

The patient recruitment rate is calculated by the ratio (total, each intervention group) of recruited patients during the study period to planned study patients (45 patients, 15 patients per each intervention group). The patient enrolment rate is calculated by dividing the total number of study patients by the total number of screening patients. Reasons for dropout will be documented, and the dropout rate is calculated and will be compared for all study patients and each intervention group.

##### Analysis of Immune Responses and Metabolites

The effects of herbal medicine after recovery from COVID-19 on immune responses and metabolites related to fatigue and cognitive dysfunction will be investigated by peripheral blood-immunologic and metabolomic analysis. The patient’s peripheral blood will be collected, 25 mL at visits 0, 4, 5, and 6. To explore the effects related to the immune response, the following experiments will be conducted:

- Single-cell RNA sequencing (scRNA-seq) for immune related gene expressions.
- Flowcytometric analysis for immunophenotyping SARS-CoV-2-specific T cell response.
- Quantitative analysis for inflammatory cytokines, and chemokines in serum sample.
- Quantitative analysis for SARS-CoV-2 antigens (spike or nucleoprotein), specific antibodies (IgG, IgM, or IgA), and neutralizing antibodies in serum samples.

Furthermore, to investigate the changes caused by herbal medicine in metabolites related to fatigue and cognitive dysfunction after recovery from COVID-19, a metabolomic analysis based on mass spectrometry will be conducted using serum sample. In the present study, using gas chromatography–mass spectrometry and liquid chromatography–mass spectrometry, metabolic changes caused by herbal medicine will be investigated using metabolite profile analysis including primary and secondary metabolites, such as alkaloids and derivatives, benzenoids, homogeneous non-metal compounds, lipids and lipid-like molecules, nucleosides, nucleotides, and analogs, organic acids and derivatives, organic nitrogen compounds, organic oxygen compounds, organo-halogen compounds, organo-heterocyclic compounds, phenylpropanoids and polyketides.

### 2.9. Safety Evaluation

For safety evaluation, adverse events, vital signs, laboratory tests, and electrocardiograms will be evaluated. Adverse events refer to all harmful and unintended symptoms or signs, including abnormal results in laboratory tests, or diseases that occurred in patients administered herbal medicine that do not necessarily have to be a causal relationship. Vital signs including blood pressure, body temperature, breathing, and heart rate, will be recorded at every visit. Electrocardiography will be performed to monitor the presence of severe heart disease, such as acute myocardial infarction and ventricular fibrillation. Laboratory tests will determine the red blood cell, hemoglobin, hematocrit, platelet, white blood cell, fasting blood sugar, BUN, creatinine, AST, ALT, and blood electrolyte (Na, K, Cl) levels, and the observed values and changes from the baseline value will be evaluated.

### 2.10. Adverse Event Reporting

The information on adverse events occurring in study patients shall be voluntarily reported by the patients or their representatives immediately, and should be confirmed through clinical examinations and interviews by the principal investigator or other researchers. The investigation for adverse events includes the date of onset and disappearance, the severity, medical measures or treatments, the causality with herbal medicine, and the progression.

#### 2.10.1. Assessment of Subjective and Objective Symptoms

The subjective and objective symptoms by adverse reaction will be investigated through medical examination by the investigator, and the severity of symptoms will be recorded following the evaluation criteria. The causality between adverse events and herbal medicine will be also evaluated.

#### 2.10.2. Assessment of Measured Tests Such as Laboratory Tests and Vital Signs

Clinically significant abnormal changes in test results, such as laboratory tests and vital signs, will be followed up until symptom improvement. Moreover, interference factors for the test will be recorded through the interview.

### 2.11. Ethics

The Institutional Review Board of Kyung Hee University Korean Medicine Hospital has approved the protocol (IRB approval numbers KOMCIRB 2020-12-002-001). This study was registered in the national clinical trial registry Clinical Research Information Service, a primary registry of the World Health Organization International Clinical Trials Registry Platform (KCT0006252).

### 2.12. Statistical Analysis

For assessing the feasibility of this study design, we will analyze the subject recruitment rate, dropout rate, and dropout reasons. The subject recruitment rate will be calculated by dividing the total number of study subjects by the total number of participants screened (%). The dropout rate will be calculated for all participants and each herbal medication group. The dropout reasons will also be assessed. For continuous variables that will be assessed to evaluate the potential clinical effectiveness of herbal medications, the standard deviation, median, minimum, and maximum values for each time point will be presented. An analysis of covariance (ANCOVA) test will be conducted for differences between herbal medication groups (*p* < 0.05). If the assumption of ANCOVA is tested and the basic assumptions such as normality and equal variance of data are violated, the Kruskal–Wallis test will be performed. Statistical analysis will be conducted using the R-software (R 4.1.2, The R Foundation, www.r-project.org, accessed on 9 August 2022) or Jamovi software (Version 1.8.0, The jamovi project, www.jamovi.org, accessed on 9 August 2022). A full analysis set will be used to analyze variables for effectiveness evaluation. Data on safety evaluation will be used with the safety set. All statistical tests, unless otherwise defined, are tested with the two-sided test, with a 5% significance level.

## 3. Discussion

With the continuously progressing global COVID-19 pandemic, medical care for the long-term effects of SARS-CoV-2 infection should be established. Long-term sequelae refer to persistent symptoms or signs for >6 months after COVID-19 recovery. According to a survey in Korea, 65.7% of responders who suffered from COVID-19 had long-term sequelae. The most common long-term sequelae are fatigue, followed by neuropsychiatric symptoms, such as concentration difficulty, amnesia, cognitive dysfunction, anxiety, and depression [13]. Furthermore, even some non-hospitalized COVID-19 patients have been reported to suffer from sequelae, such as fatigue, shortness of breath, cognitive dysfunction, and stress/anxiety at least once within 30 days or longer [14]. These neuropsychiatric symptoms and signs, such as fatigue and cognitive dysfunction after COVID-19, are considered a category of chronic fatigue syndrome/myalgic encephalomyelitis (CFS/ME). Indeed, several viruses are associated with CFS/ME, such as hepatitis C virus, human immunodeficiency virus, and Epstein–Barr virus [15]. These neurological complications have been suggested to be caused by a disturbance in neuroimmune regulation. The reduced anti-inflammatory cytokine (IL-10) level observed in the cerebrospinal fluid (CSF) may be related to neurological complications in patients with CFS/ME [16,17], and abnormal cytokine levels due to immune responses in SARS-CoV-2 infection have been reported to potentially contribute to the development of neurological complications, such as CFS/ME [18]. Furthermore, autopsy demonstrates SARS-CoV-2 may cause neuropathological lesions both in the parenchyma and vessels of the brain [19]. It also supports the theory that SARS-CoV-2 invades the choroid plexus epithelium, thereby resulting in leakage of the blood-CSF barrier [15]. In contrast, the finding of paucity of neuroinflammatory changes and absence of SARS-CoV-2 viral RNA in CSF of COVID-19 patients with neurological complications does not provide evidence of neuroinflammation in pathogenesis of SARS-CoV-2 [20]. Hence, the exact cause of neuropsychiatric sequelae in COVID-19 is still obscure, a conventional treatment for the sequelae has not yet been established, and symptomatic treatment and lifestyle management are being implemented. As the prevalence of these sequelae is expected to further increase as the spread of COVID-19 continues, related further studies are necessary.

Bojungikgi-tang (CV1), herbal medicine that will be administered to the fatigue group in the present study, has been investigated as the most frequently prescribed herbal medicine for the treatment and management of chronic fatigue by KMDs [21], and our systematic literature review reveals that Bojungikgi-tang has a significant effect in alleviating the symptoms of CFS compared to health supplements or symptomatic treatments. [22]. In addition, Kyungok-go (CV2), to be prescribed to another fatigue group, is used to improve physical fatigue, malaise, and residual symptoms after disease in KM clinical practice, and is one of the herbal medicines most prescribed to patients in the COVID-19 KM telemedicine program [23]. An animal study also provided evidence that Kyungok-go shows a significant improvement in exercise capacity and anti-fatigue properties by reducing the lactate level, enhancing the blood glucose level, and increasing glycogen in the skeletal muscles [24]. Cheonwangbosim-dan (CV3), the herbal medicine to be administered for cognitive dysfunction in this study, has reportedly been effective for memory improvement in an Alzheimer’s disease mouse model by inhibiting acetylcholinesterase, reducing hippocampal infarction, and suppressing proinflammatory cytokine production such as IL-1β and TNF-α [25]. Therefore, the results of these studies indicate that the herbal medicines to be administered in the present study have the possibility to mitigate fatigue and cognitive dysfunction after COVID-19 recovery.

A recent study on patients with convalescent COVID-19 found that most patients had SARS-CoV-2-specific IgG seropositivity until >7 months after symptom onset. However, many of their plasma samples showed insufficient neutralizing activity to maintain a protective barrier [26], and the development of neutralizing antibodies may be related to the activation of SARS-CoV-2-specific T cells and NK cells [27]. Therefore, the maintenance of long-term immunity to SARS-CoV-2 may demand a synergistic humoral and cellular immune response, and medical management is highly required. Moreover, the metabolic state of patients is important for immune response and prognosis in COVID-19. Indeed, abnormal metabolic conditions, such as obesity and diabetes, have been associated with increased mortality and long-term sequelae in COVID-19 by affecting immune responses due to increased inflammation and impaired energy generation [28]. A previous metabolomic study of patients with COVID-19 suggested that 204 metabolites might be correlated with the severity of COVID-19, and metabolic pathways and plasma metabolites are associated with inflammation and immune activation. These metabolomic data show that >100 metabolites including amino acids and their derivatives and >100 lipids including glycerophospholipids, sphingolipids, and fatty acids are decreased in the patient sera [29]. Furthermore, CFS/ME is a consequence of hypometabolic response to environmental stress, and several metabolic pathways are shown to be impaired with abnormal metabolite levels in the plasma of patients with CFS/ME [30,31]. Therefore, metabolic function and immune response caused by changes due to COVID-19 may be responsible for the sequelae, and in the present study we will investigate the effects of herbal medicine on changes in COVID-19 to provide an insight into the preparation of medical management for post-acute sequelae of SARS-CoV-2 infection.

Considering this situation, in the present study, we will administer the herbal medicines for 12 weeks maximum to patients who have fatigue or cognitive dysfunction after COVID-19 recovery, and will explore the symptom improvement associated with herbal medicine and its effect on metabolites and immune response in the peripheral blood sample. The present study might suggest the feasibility of clinical studies for herbal medications to treat post-acute sequelae following COVID-19 recovery; nevertheless, further randomized-controlled trials with sufficient sample size should be executed to establish clinical evidence on their effectiveness and safety.

## Figures and Tables

**Table 1:** Schedule summary.

Visit	0	1	2	3	4	5	6	UV *
Week	−7	1	5	9	13	25	37	-
Visit Window	-	2	±7	±7	±7	±7	±7	-
Informed consent for the study	●							
Demographic information	●							
Participation in other clinical trials	●							
Medical history	●							
Confirmation of diagnosis of COVID-19 infection	●							
Medication history	●							
Vital signs	●	●	●	●	●	●	●	●
Electrocardiogram	●				●			●
Laboratory test	●				●			●
Blood collection for immune response and metabolite analysis	●				●	●	●	●
Pregnancy **	●							
Evaluation of fatigue or brain fog	●							
CIS	●		●	●	●	●	●	●
VAS (0–100) score for fatigue or brain fog	●		●	●	●	●	●	●
Inclusion/exclusion criteria	●							
KM syndrome differentiation		●						
Prescription of herbal medicine ***		●	○	○				
Drug adherence			●	●	●			●
Check for combination therapy			●	●	●	●	●	●
ChFQ		●	●	●	●	●	●	●
EQ-5D-5L		●	●	●	●	●	●	●
PSQI-K		●	●	●	●	●	●	●
K-MoCA		●	●	●	●	●	●	●
CFQ		●					●	●
BDI		●	●	●	●	●	●	●
Digit span test in K-WAIS (DF, DB, and DF-DB)		●			●	●	●	
K-BNT-15		●			●	●	●	
Adverse events check		●	●	●	●	●	●	●

●, check on visit; ○, if applicable * UV, unscheduled visit (), ** only fertile women, *** Evaluation for the continuation of herbal medicine administration at 5th and 9th weeks.

**Table 2:** KM syndrome differentiation for allocation to treatment group.

	Fatigue		Cognitive Dysfunction
Code	CV1	CV2	CV3
Syndrome differentiation classification	Spleen-lung qi deficiency pattern	Dual deficiency of qi and yin pattern	Heart yin deficiency heat pattern
Symptom	Fatigue, appetite loss, cold sweat, shortness of breath, chest tightness, anxiety and others	Fatigue, dry cough and others	Forgetfulness, fever, insomnia, heart palpitation, stomatitis, tongue needles and others
Tongue diagnosis	Pale tongue, thin white fur	Dry mouth, dry tongue	Red tongue and low tongue coated
Pulse diagnosis	Vacuous, large, weak pulse/surging, large pulse	Fine pulse/vacuous, weak pulse	Fine, rapid pulse
Urine/feces	Difficult stool to pass/sloppy stool	Dry stool	Inhibited stool/sloppy stool

## Data Availability

This study was registered in the national clinical trial registry Clinical Research Information Service, which is a primary registry of the World Health Organization International Clinical Trials Registry Platform (KCT0006252).
